# Comparative efficacy of low-dose versus standard-dose azithromycin for patients with yaws: a randomised non-inferiority trial in Ghana and Papua New Guinea

**DOI:** 10.1016/S2214-109X(18)30023-8

**Published:** 2018-02-16

**Authors:** Michael Marks, Oriol Mitjà, Christian Bottomley, Cynthia Kwakye, Wendy Houinei, Mathias Bauri, Paul Adwere, Abdul A Abdulai, Fredrick Dua, Laud Boateng, James Wangi, Sally-Ann Ohene, Regina Wangnapi, Shirley V Simpson, Helen Miag, Kennedy K Addo, Laud A Basing, Damien Danavall, Kai H Chi, Allan Pillay, Ronald Ballard, Anthony W Solomon, Cheng Y Chen, Sibauk V Bieb, Yaw Adu-Sarkodie, David CW Mabey, Kingsley Asiedu, Additional members of the yaws clinical trial study team, Nsire Agana, Edwin Ampadu, Kwame Amponsah-Achiano, Asare Bediako, Michael Biredu, Kyei Faried, Ahmed Iddrisu, Nana Konama Kotey, George Nana Yaw Yeboah, Philip El-Duah, Richard Phillips, Fred Binka, Frank Nyonator, Anthony Zunuo, Mercy Mawufenya Ackumey, Ivy Amanor, Christian Bonsu, Sieghard Frischmann, Patrick Lammie, Diana Martin, Tun Ye, Eva Christophel, Lasse Vestergard, Alexandre Tiendrebeogo, Quique Bassat, Yazid Abdad, Henson Dima, Bethuel Kotty, Kaiok Mamore, Walerius Manup, Benson Olowau, Enoch Omane Agyei, David Agyemang, Ebenezer Padi Ako, Prince Antwi, Jane Darko, Ophelia Oppong Darko, Phyllis Darko, Bertha Duodu, Daniel Jabasi, Fuseini L Karim, Obed Kofi Koomson, Bernard Antwi Larbi, John Nartey, Ransford Tamatey, Benjamin Yirenkyi, Mercy Arhin, Frank Biney, Juliana Oparebea Danso, Martin Adjei Dei, Moses Djan, Samuel Sasu, Brefo Antwi Solomon, Victor Torvinya, Hagar Amankwaah, James Baffoe, Moses Djan, Lydia Keteku, Kofi Kondobala, Rita Dede Lomotey, Augustina Addy Nartey, Paul Oppong, Millicent Aba Quainoo, Theophilus Abotsi, Dzigbordi Agbeshie, Amos Ameamu, Paul Angwaawie, Rose Ayibor, Margaret Mwingmendeli, John Nakodja, Amatus Nambagyira, Dominic Nanga, Nicholas Tetteh, Augustine Wanaom, Data Safety and Monitoring Board, Anthony Danso-Appiah, Christin Johnson, Jean-Christophe Luthi, Chandrakant Revankar, Peter Smith, Ymkje Stienstra

**Affiliations:** Clinical Research Department, Faculty of Infectious and Tropical Diseases; London School of Hygiene & Tropical Medicine, London, UK; Hospital for Tropical Diseases, London, UK; Barcelona Institute for Global Health, University of Barcelona, Barcelona, Spain; Lihir Medical Centre, International SOS, Newcrest Mining, Lihir Island, Papua New Guinea; Clinical Research Department, Faculty of Infectious and Tropical Diseases and MRC Tropical Epidemiology Group, Faculty of Epidemiology and Public Health; Ghana Health Services, Accra, Ghana; Department of Health, Port Moresby, Papua New Guinea; Department of Health, Port Moresby, Papua New Guinea; Ghana Health Services, Accra, Ghana; Ghana Health Services, Accra, Ghana; Ghana Health Services, Accra, Ghana; Ghana Health Services, Accra, Ghana; World Health Organization Country Office, Port Moresby, Papua New Guinea; World Health Organization Country Office, Accra, Ghana; Papua New Guinea Institute of Medical Research, Goroka, Papua New Guinea; Noguchi Memorial Institute for Medical Research, Accra, Ghana; Department of Health, Port Moresby, Papua New Guinea; Noguchi Memorial Institute for Medical Research, Accra, Ghana; Kwame Nkrumah University of Science and Technology, Kumasi, Ghana; Molecular Diagnostics and Typing Laboratory, Laboratory Reference and Research Branch, Division of STD Prevention; Molecular Diagnostics and Typing Laboratory, Laboratory Reference and Research Branch, Division of STD Prevention; Molecular Diagnostics and Typing Laboratory, Laboratory Reference and Research Branch, Division of STD Prevention; Molecular Diagnostics and Typing Laboratory, Laboratory Reference and Research Branch, Division of STD Prevention Center for Global Health; Centers for Disease Control and Prevention, Atlanta, GA, USA; Department of Control of Neglected Tropical Diseases, World Health Organization, Geneva, Switzerland; Molecular Diagnostics and Typing Laboratory, Laboratory Reference and Research Branch, Division of STD Prevention; Centers for Disease Control and Prevention, Atlanta, GA, USA; Department of Control of Neglected Tropical Diseases, World Health Organization, Geneva, Switzerland Department of Public Health, National Department of Health, Waigani, Papua New Guinea; Kwame Nkrumah University of Science and Technology, Kumasi, Ghana; Clinical Research Department, Faculty of Infectious and Tropical Diseases; London School of Hygiene & Tropical Medicine, London, UK; Hospital for Tropical Diseases, London, UK; Centers for Disease Control and Prevention, Atlanta, GA, USA; Department of Control of Neglected Tropical Diseases, World Health Organization, Geneva, Switzerland; Ghana Health Service, Ghana; Komfo Anokye Teaching Hospital, Ghana; University of Health and Allied Sciences, Ghana; University of Ghana, Ghana; Noguchi Memorial Institute for Medical Research, University of Ghana, Ghana; MAST Diagnostics, Germany; Centers for Disease Control and Prevention, GA, USA; World Health Organization, Philippines; World Health Organization, Congo; Barcelona Institute for Global Health, Spain; Papua New Guinea Institute of Medical Research, Papua New Guinea; Field teams in Ghana: Ayensuano; West Akim; Upper West Akim; Nkwanta North; School of Public Health, University of Ghana, Legon, Ghana; Fondation Raoul Follereau, Lyon, Benin; University Institute of Social and Preventive Medicine, Lausanne, Switzerland and Centre Hospitalier Universitaire Vaudois [CHUV], Lausanne, Switzerland; independent consultant about neglected tropical diseases, North Brunswick, NJ, USA; London School of Hygiene & Tropical Medicine, London, UK; University Medical Center Groningen, Groningen, Netherlands

## Abstract

**Background:**

A dose of 30 mg/kg of azithromycin is recommended for treatment of yaws, a disease targeted for global eradication. Treatment with 20 mg/kg of azithromycin is recommended for the elimination of trachoma as a public health problem. In some settings, these diseases are co-endemic. We aimed to determine the efficacy of 20 mg/kg of azithromycin compared with 30 mg/kg azithromycin for the treatment of active and latent yaws.

**Methods:**

We did a non-inferiority, open-label, randomised controlled trial in children aged 6–15 years who were recruited from schools in Ghana and schools and the community in Papua New Guinea. Participants were enrolled based on the presence of a clinical lesion that was consistent with infectious primary or secondary yaws and a positive rapid diagnostic test for treponemal and non-treponemal antibodies. Participants were randomly assigned (1:1) to receive either standard-dose (30 mg/kg) or low-dose (20 mg/kg) azithromycin by a computer-generated random number sequence. Health-care workers assessing clinical outcomes in the field were not blinded to the patient’s treatment, but investigators involved in statistical or laboratory analyses and the participants were blinded to treatment group. We followed up participants at 4 weeks and 6 months. The primary outcome was cure at 6 months, defined as lesion healing at 4 weeks in patients with active yaws and at least a four-fold decrease in rapid plasma reagin titre from baseline to 6 months in patients with active and latent yaws. Active yaws was defined as a skin lesion that was positive for *Treponema pallidum* ssp *pertenue* in PCR testing. We used a non-inferiority margin of 10%. This trial was registered with ClinicalTrials.gov, number NCT02344628.

**Findings:**

Between June 12, 2015, and July 2, 2016, 583 (65·1%) of 895 children screened were enrolled; 292 patients were assigned a low dose of azithromycin and 291 patients were assigned a standard dose of azithromycin. 191 participants had active yaws and 392 had presumed latent yaws. Complete follow-up to 6 months was available for 157 (82·2%) of 191 patients with active yaws. In cases of active yaws, cure was achieved in 61 (80·3%) of 76 patients in the low-dose group and in 68 (84·0%) of 81 patients in the standard-dose group (difference 3·7%; 95% CI −8·4 to 15·7%; this result did not meet the non-inferiority criterion). There were no serious adverse events reported in response to treatment in either group. The most commonly reported adverse event at 4 weeks was gastrointestinal upset, with eight (2·7%) participants in each group reporting this symptom.

**Interpretation:**

In this study, low-dose azithromycin did not meet the prespecified non-inferiority margin compared with standard-dose azithromycin in achieving clinical and serological cure in PCR-confirmed active yaws. Only a single participant (with presumed latent yaws) had definitive serological failure. This work suggests that 20 mg/kg of azithromycin is probably effective against yaws, but further data are needed.

## Introduction

Yaws, a neglected tropical disease caused by *Treponema pallidum* ssp *pertenue*, remains an important public health problem in remote communities of 14 countries in Africa, Asia, and the western Pacific.^1,2^ Yaws is closely genetically related to syphilis, but these diseases can be differentiated on the basis of clinical features and through molecular testing. Yaws predominantly affects children younger than 15 years and transmission occurs by direct contact with a person with an early infectious lesion. The early lesions of primary and secondary yaws predominantly manifest as chronic cutaneous ulcers and papillomas.^1^ A serological diagnosis of yaws requires the detection of both treponemal and non-treponemal antibodies.^1^ Alongside clinical improvement, non-treponemal titres are expected to fall following treatment with effective antibiotics. More recently, point-of-care serological tests and PCR assays have become available to aid diagnosis.^3–6^ If left untreated, yaws can progress to destructive lesions of the bone and soft tissues.

Long-acting injectable penicillin has been the mainstay of yaws treatment since WHO and the United Nations Children’s Fund led campaigns in the 1950s. In 2012, a randomised controlled trial (RCT)^7^ in Papua New Guinea showed that a single dose of 30 mg/kg of azithromycin (maximum 2 g) was non-inferior to benzathine benzylpenicillin for the treatment of yaws. Following this finding, which was later confirmed by a similar study in Ghana,^8^ WHO developed a new eradication strategy for yaws that involved mass treatment of the community with azithromycin.^9^ Pilot studies^10^ suggest that azithromycin mass treatment is very effective at reducing the prevalence of infection and disease.

Mass treatment of the community with azithromycin is also key to WHO’s SAFE strategy for the elimination of trachoma as a public health problem;^11^ however, the dose of azithromycin used in trachoma programmes (20 mg/kg, maximum 1 g) is lower than the recommended dose for treatment of yaws. This difference in recommendations is potentially problematic because, in areas where trachoma and yaws are co-endemic,^12^ although mass treatment for trachoma elimination programmes might aid efforts to eradicate yaws if the dose used is effective against yaws, it could have negative consequences if the lower dose for trachoma treatment is subtherapeutic and results in more rapid selection of macrolide-resistant strains of *T pallidum* ssp *pertenue*. Azithromycin resistance spread rapidly in syphilis, caused by the genetically related *T pallidum* ssp *pallidum*.^13–15^ Establishing definitive data on the efficacy of low-dose azithromycin has therefore been identified as a priority by the International Task Force for Disease Eradication and partners.^16,17^ Studies^18,19^ in the Solomon Islands have shown that mass administration of azithromycin to eliminate trachoma has significant effects on the prevalence of both active and latent yaws; however, to date, no formal trials have compared the efficacy of the two doses. If a 20 mg/kg dose was shown to effectively treat yaws, this finding could potentially reduce both the costs of eradication and the incidence of adverse events associated with mass drug administration. This result might also allow synergistic treatment of yaws via trachoma elimination programmes (or vice versa) in areas where the diseases are co-endemic.^16^

Although a lower dose of azithromycin might be effective at treating the relatively more metabolically active treponemes in a skin lesion, it might be less effective in latent infections, in which the bacteria are postulated to be less metabolically active.^1^ Since treatment of both active and latent yaws is crucial for yaws eradication,^20^ it is important that the efficacy of treatment with a low dose of azithromycin be established both for active and latent yaws.

We therefore conducted an RCT in Ghana and Papua New Guinea, the countries that report the most cases of yaws globally,^2^ to determine whether the efficacy of low-dose azithromycin was non-inferior to standarddose azithromycin in the treatment of yaws.

## Methods

### Study design and participants

**Figure 1:**
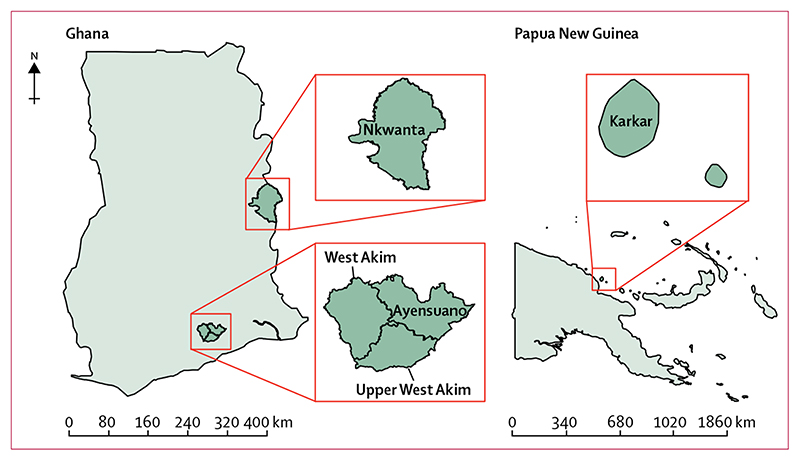
Districts of study recruitment in Ghana and Papua New Guinea

We did a randomised, controlled, open-label noninferiority trial in four districts of Ghana and one district of Papua New Guinea (figure 1). Participants were recruited from schools in Ghana and schools and the community in Papua New Guinea and they were treated at their location of recuitment. None of these districts had previously received mass treatment with azithromycin for trachoma, but West Akim had received some mass treatment for yaws in 2013. Before study commencement, district study teams and officials of the national yaws eradication programmes of each country ran educational sessions to inform the selected communities about the study.

We identified children aged 6–15 years (the age range in which yaws incidence peaks) with a clinical lesion that was consistent with infectious primary or secondary yaws, which comprised at least one ulcerative lesion or papilloma, as described in the WHO yaws booklet.^21^ We collected a finger-prick blood sample from each child. A rapid diagnostic test (dual path platform [DPP] syphilis screen and confirm assay; Chembio Diagnostics, Medford, NY, USA) was done, which detects both treponemal and non-treponemal antibodies.^22^ Children with a clinically suspicious lesion and a dual-positive rapid diagnostic test (positive for both treponemal and non-treponemal tests; appendix 1) met the inclusion criteria and were invited to enrol in the study. Children were excluded if they had a known allergy to azithromycin or macrolides, had received treatment with an antibiotic effective against *T pallidum* in the past 3 months, or had another contraindication to treatment with the study drug.

Before study commencement, teams underwent standardised training in clinical diagnosis of yaws, performance of the rapid diagnostic test, collection of blood and lesion samples, management of adverse events, and completion of documentation in line with the Good Clinical Practice guidelines.

Written informed consent was obtained from the parent or guardian of each participant. Consent was also obtained from children who could provide it. The study was approved by the ethics committees of WHO (RPC 720), the London School of Hygiene & Tropical Medicine (London, UK; 8832), the US Centers for Disease Control and Prevention (CDC; Atlanta, GA, USA; 6746/7285), the Ghana Health Service (Accra, Ghana; 13/11/14), the Papua New Guinea National Department for Health (Port Moresby, Papua New Guinea; MRAC 14.31), and the Papua New Guinea Institute of Medical Research (Goroka, Papua New Guinea; 1504). The study was done in accordance with the Declaration of Helsinki and was monitored by an independent Data and Safety Monitoring Board.

### Randomisation and masking

Eligible children were randomly assigned to either a low-dose (20 mg/kg, maximum 1 g) or standard-dose (30 mg/kg, maximum 2 g) group. Randomisation was performed in blocks of four, by use of a computergenerated random number sequence that was generated by the trial statistician (CB) at the London School of Hygiene & Tropical Medicine. Allocation was concealed from investigators by use of opaque, sealed, sequentially numbered envelopes that were opened after the study team had enrolled a participant. Health-care workers assessing clinical outcomes in the field were not masked to the patient’s treatment group but patients and investigators performing statistical or laboratory analyses of samples were masked to treatment allocation. Study allocations are listed in appendix 2.

### Procedures

Children were seen at baseline for enrolment, initial data collection, and treatment, and again for follow-up at 4 weeks and at 6 months. At each visit, children received a standardised skin examination, during which the type and location of any yaws-like lesions were recorded. In participants with more than one yaws-like lesion, the largest lesion was identified as the lesion-of-interest for sample collection and determination of healing at followup. Photographs were taken of the lesion-of-interest and the DPP results at each timepoint. All clinical data were collected directly into smartphones with the LINKS software package.^23^

A 5 mL sample of serum was obtained from all enrolled participants at baseline and at the 6-month follow-up. Additionally, at baseline, a single swab sample was collected from the target lesion and placed into 1·2 mL of AssayAssure transport medium (Thermo Fisher Scientific, Waltham, MA, USA) as previously described.^3^ At 4 weeks, if there had been no healing of the lesion, a repeat swab sample was obtained and the participant was offered treatment with benzathine benzylpenicillin. At 6 months, if the lesion was still not fully healed, a repeat swab sample was obtained and the participant was offered treatment with benzathine benzylpenicillin, or referred to the local health facility for further management, or both.

Serum and lesion samples were transported on dry ice to the CDC laboratories in Atlanta, GA, USA. Serum samples were tested with the Serodia *T pallidum* passive particle agglutination test (Fujirebio Diagnostics, Malvern, PA, USA) and a quantitative rapid plasma reagin (RPR) test (Alere, Waltham, MA, USA). Because *Haemophilus ducreyi* commonly causes skin lesions that can be clinically difficult to distinguish from the lesions of yaws infections, and Buruli ulcer is co-endemic in both countries,^4,24,25^ two real-time multiplex PCR assays for the presence of *T pallidum* ssp *pertenue, H ducreyi*, and *Mycobacterium ulcerans* were done on swab samples, as previously described.^3,24^ The *T pallidum* PCR used has previously been shown to have an analytical sensitivity of ten copies per sample.^3^ The multiplex PCR assays also incorporated an amplification of the human RNA polymerase gene to confirm the integrity of samples and the absence of PCR inhibition. Additionally, all samples were tested by use of an additional multiple PCR for mutations associated with azithromycin resistance.^26^ The use of PCR in the current study allowed delineation of the effect of the two dosing strategies in both participants with active yaws (positive in a PCR analysis for *T pallidum* ssp *pertenue* in a lesion) and in participants with presumed latent yaws and a different cause of the current skin lesion (negative in a PCR analysis for *T pallidum* ssp *pertenue* in a lesion).

All participants were treated with azithromycin, which was administered as a single oral dose. WHO purchased azithromycin from Medopharm (India) and DPP syphilis screen and confirm rapid diagnostic tests from Chembio Diagnostics. Other study materials were provided by WHO and CDC. A small snack was provided so that individuals could eat before treatment. The study team directly observed treatment. Participants were observed for 1 hour following treatment to monitor for immediate adverse events. If vomiting occurred within this time, the child was treated again. Children who did not meet inclusion criteria for the study also received a standard 30 mg/kg dose of azithromycin, in accordance with WHO recommendations, and their ulcers were dressed. Any side-effects were monitored and managed by the study team or patients were referred to the district hospital for further treatment. Immediate adverse events were documented at the time of drug administration while the participant remained under observation. Participant-reported adverse events were documented at the 4-week follow-up visit.

### Outcomes

The primary endpoint was cure at 6 months of treatment. Cure was a composite outcome of both clinical cure and serological cure. Clinical cure was defined as complete or partial resolution of the lesion by 4 weeks. Clinical failure was defined as no evidence of healing at the 4-week follow-up visit (appendix). Serological cure was defined as a decrease in the quantitative RPR titre between baseline and 6 months of greater than 4-fold (eg, from 1:32 to 1:8) or RPR seroreversion by 6 months. The primary endpoint was measured in participants with both serologically and PCR-confirmed yaws.

Serological cure and clinical cure were analysed separately as secondary outcomes. Serological failure was defined as an increase in RPR titre between baseline and 6 months of at least 4-fold (eg, from 1:4 to 1:16). Participants with a negative RPR at baseline were excluded from the analyses of both the composite primary outcome and the secondary outcomes because serological outcomes could not be assessed in this patient group. Participants with a missing baseline or 6-month RPR result or a missing baseline lesion PCR result were also excluded from all analyses.

### Statistical analysis

The study was designed to assess whether treatment with 20 mg/kg of azithromycin (maximum 1 g) was non-inferior to treatment with 30 mg/kg (maximum 2 g) of azithromycin for the primary outcome in participants who were PCR-positive for *T pallidum* ssp *pertenue* at baseline. A prespecified non-inferiority margin of 10% was used. Non-inferiority was defined as an upper bound of the two-sided 95% CI for the difference in proportions (ie, proportion of participants in the 30 mg/kg treatment group who achieved cure minus the proportion of participants in the 20 mg/kg treatment group who achieved cure) of less than or equal to 10%. This margin was selected to reflect the maximum difference in efficacy that would allow the lower dose to remain acceptable for use in yaws eradication efforts. For secondary outcomes, non-inferiority was also assessed with the use of a two-sided 95% CI for the between-group difference. All confidence intervals were calculated by use of the Agresti-Caffo method.^27^

The primary analysis was restricted to participants with lesion samples that were positive by PCR for *T pallidum* ssp *pertenue* at baseline. We did pre-planned secondary analyses of this population (patients who were PCR-positive for *T pallidum* ssp *pertenue* at baseline) by country of recruitment. Further pre-planned analyses were done on the following secondary study populations: (1) all study participants, regardless of baseline PCR status; and (2) participants who had a negative *T pallidum* ssp *pertenue* PCR result at baseline. Among participants with a negative *T pallidum* ssp *pertenue* PCR result at baseline, we separately evaluated the proportion achieving clinical cure in patients who tested positive for *H ducreyi* DNA and in patients who tested negative for *H ducreyi* DNA in their lesion at baseline.

To investigate the effects of missing data, we did a sensitivity analysis using multiply imputed data on the primary analysis population of participants with *T pallidum* ssp *pertenue* DNA detected at baseline. The imputation model included all baseline and outcome variables, and was implemented by use of the method of multiple chained equations.^28^

Assuming 95% efficacy of standard treatment, a non-inferiority margin of 10%, a type I error rate of 5%, and 10% loss to follow-up, we calculated that a total of 220 PCR-confirmed cases of yaws (ie, 110 cases per study group) would be required for the primary analysis. Based on PCR data collected in previous studies, we calculated that 524 individuals with clinically suspicious yaws lesions and a positive point-of-care serological test would be needed to achieve this sample size. Analyses were performed in Stata version 14.0 (StataCorp, College Station, TX, USA). This trial is registered with ClinicalTrials.gov, number NCT02344628.

### Role of the funding source

The funder of the study had no role in study design, data collection, data analysis, data interpretation, or writing of the report. The corresponding author had full access to all the data in the study and had final responsibility for the decision to submit for publication.

## Results

**Figure 2:**
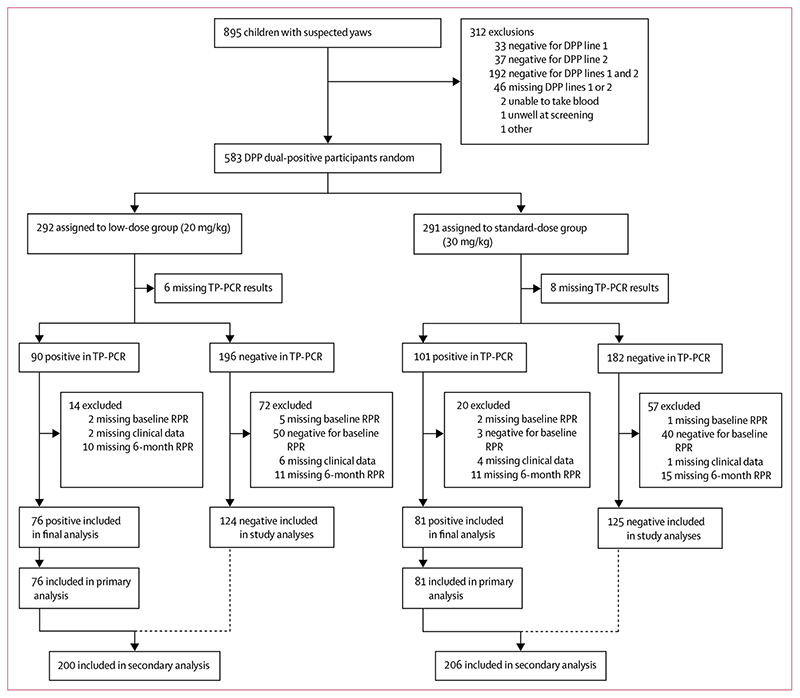
Trial profile DPP=dual path platform, a syphilis screen and confirm assay. TP-PCR= *Treponema pallidum* PCR. RPR=rapid plasma reagin.

Between June 12, 2015, and July 2, 2016, 895 children with clinically suspected yaws were screened for enrolment. Of these, 583 (65·1%) had a positive DPP point-of-care test and were enrolled (figure 2). 400 (68·6%) participants were enrolled in Ghana and 183 (31·4%) were enrolled in Papua New Guinea. 292 participants were randomly assigned to the low-dose group and 291 to the standarddose group (figure 2). Baseline clinical and serological characteristics were similar between the two groups: the median age of participants was 10 years (IQR 8–12) and 412 (70·7%) participants were male (table 1).

Baseline PCR was positive for *T pallidum* ssp *pertenue* alone in 161 participants (27·6%; 75 in the low-dose group and 86 in the standard-dose group) and for both *T pallidum* ssp *pertenue* and *H ducreyi* in 30 participants (5·1%; 15 in each group). These two groups constituted the population for the primary study analysis. The remaining 392 participants were included in secondary analyses; they comprised 150 (25·7%) participants with only *H ducreyi*-positive lesions by PCR (75 in each group), 228 (39·1%) participants with negative PCR results for all pathogens tested for (121 in the low-dose group and 107 in the standard-dose group), and 14 (2·4%) participants whose baseline PCR data were missing (six in the low-dose group and eight in the standard-dose group). No samples tested positive for *M ulcerans*.

In the primary analysis of participants with PCR-confirmed active yaws (table 2) the non-inferiority criterion was not met for the composite primary outcome of clinical and serological cure at 6 months. The primary outcome was achieved in 68 (84·0%) of 81 participants in the standard-dose group and 61 (80·3%) of 76 participants in the low-dose group (absolute difference 3·7%, 95% CI −8·4 to 15·7%). The proportion achieving clinical cure did not differ significantly between groups. The proportion achieving serological cure was slightly higher in the standard-dose group, but this difference was not significant (table 2).

**Figure 3:**
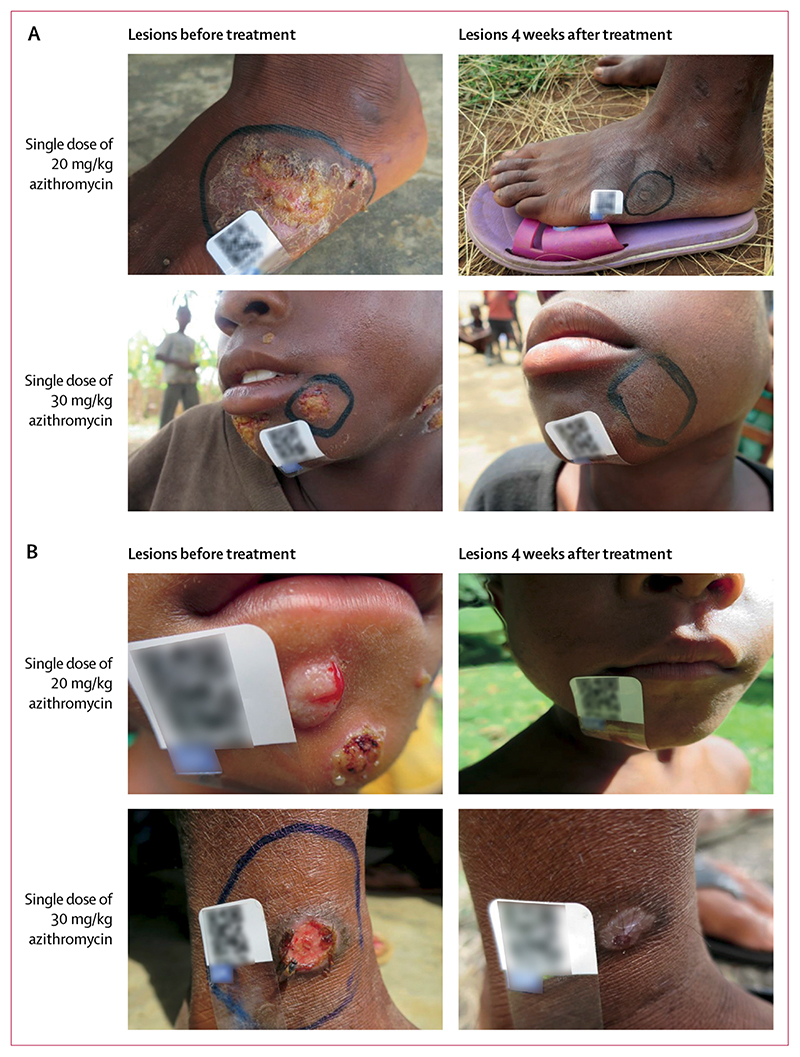
Clinical outcomes of treatment Clinical healing of lesions from baseline to 4 weeks following treatment with either low-dose or standard-dose azithromycin in (A) Papua New Guinea and (B) Ghana.

Clinical healing of lesions in both groups is shown in figure 3. Of the 28 participants who did not achieve serological cure at 6 months (15 in the low-dose group and 13 in the high-dose group), none met the definition of serological failure: all had a non-significant RPR change at 6 months, of which 18 showed only a 2-fold decrease in RPR titre (seven in the low-dose group and 11 in the standard-dose group), seven showed no change in RPR titre (six in the low-dose group and one in the standard-dose group), and three showed a 2-fold increase in RPR titre (two in the low-dose group and one in the standard-dose group). The proportion of participants achieving clinical cure did not significantly differ countries, nor did the proportion achieving serological cure (table 2; p=0·223). Clinical outcomes were similar when assessed at 4 weeks and 6 months (appendix).

No participant with clinical treatment failure had detectable *T pallidum* ssp *pertenue* DNA in any lesion at follow-up. No mutations associated with azithromycin resistance in *T pallidum* were detected at either baseline or follow-up.

In the secondary analysis, in which data from all participants were analysed regardless of baseline PCR status (ie, participants included in the primary analysis who were either positive or negative for *T pallidum* by PCR), the primary outcome was achieved in 132 (64·1%) of 206 patients assigned to the standard-dose group compared with 134 (67·0%) of 200 patients assigned to the low-dose group; this result met the non-inferiority criterion (absolute difference -3%, 95% CI -12·1 to 6·3). In this secondary analysis, the proportion of participants achieving clinical cure and the proportion of patients achieving serological cure were similar in the low-dose group (table 3). The serological cure rate was higher in participants with a baseline RPR concentration of 1:16 or greater (72·0% in patients with a baseline RPR ≥1:16 *vs* 54·2% in patients with a baseline RPR <1:16, p<0·001; appendix). Of 139 patients who did not achieve serological cure at 6 months (66 in the low-dose group and 73 in the high-dose group), only one participant (in the standarddose group) had a four-fold RPR titre increase consistent with definitive serological failure. Of these participants, 77 (34 from the low-dose group and 43 from the standarddose group) showed only a two-fold decrease in RPR titre, 47 (26 from the low-dose group and 21 from the standarddose group) showed no change to their RPR titre, and 14 (six from the low-dose group and eight from the standard-dose group) had a two-fold increase in RPR titre. In the secondary analysis, the proportion of participants with *T pallidum* ssp *pertenue* PCR-negative lesions at baseline who achieved cure was higher in the low-dose group, meeting the non-inferiority criterion (table 3). The proportion of patients in both study groups who achieved clinical cure was high, regardless of whether *H ducreyi* was detected at baseline or not (table 3).

In the pre-planned analysis on the basis of multiply imputed data, the proportions of participants achieving cure were 80·8% in the standard-dose group and 80·2% in the low-dose group (absolute difference 0·5%, 95% CI -12·2 to 13·3).

There were no serious adverse events reported in response to treatment in either group. In the active surveillance data collected from participant reports at 4 weeks, the most commonly reported adverse event was gastrointestinal upset, with no significant difference in frequency between groups (2·7% *vs* 2·7%; eight in each group; appendix).

We did a post-hoc analysis of 93 individuals with a negative RPR at baseline who were excluded from the main analyses. This group included three participants in the standard-dose group whose lesions at baseline were *T pallidum* ssp *pertenue* PCR-positive. Of the 93 participants, 82 (88%) participants had a 6-month RPR result available. Four (5%) participants were found to have definitive evidence of seroconversion (an RPR titre of 1:2 or higher) at 6 months (two from each group), of whom two had a *T pallidum* ssp *pertenue* PCR-positive lesion at baseline. A further seven (9%) participants had a conversion from a negative to undiluted positive RPR (four from the low dose group and three from the standard-dose group), which might represent false-positives, and the remaining 71 (87%) remained RPR-negative at 6 months (39 from the low-dose group and 32 from the standard-dose group).

## Discussion

In this randomised, controlled, non-inferiority trial, low-dose (20 mg/kg) azithromycin did not meet the prespecified non-inferiority margin compared with standard-dose (30 mg/kg) azithromycin in achieving clinical and serological cure in PCR-confirmed active yaws. The fact that we did not find non-inferiority of this dosage reflects wide confidence intervals around cure frequencies, which arose from the smaller than anticipated number of PCR-positive cases, and the lower than anticipated proportion of participants achieving serological cure. Despite this, low-dose azithromycin achieved a clinical cure rate of 100% (identical to the standard dose), which met the non-inferiority criterion, and a point estimate for serological cure of 80%, which, despite not meeting the non-inferiority criterion, did not differ significantly from standard-dose treatment (84%). Additionally, our pre-planned secondary analyses of all individuals regardless of PCR results, and of participants who were PCR-negative, both suggested non-inferiority of low-dose treatment. These findings, considered together, imply that standard-dose and low-dose azithromycin are likely to be equally effective for patients with yaws. Unfortunately, we were unable to show this unequivocally in participants with active yaws in the current study.

The overall proportion of participants achieving serological cure at 6 months was lower than anticipated. As in previous studies^7,8^ comparing benzathine benzylpenicillin to azithromycin for treatment of yaws, the proportion achieving serological cure at 6 months was lower in participants enrolled in Ghana than in those enrolled in Papua New Guinea, although this difference did not reach statistical significance. Two-thirds of our participants were enrolled in Ghana, which could partly explain the lower overall proportion of participants achieving serological cure at 6 months. Geographical variation in the outcome of randomised trials is well recognised, although it is unclear to us why serological cure rates might differ between these two settings. Strain differences, host immune responses, and random chance could all conceivably contribute. Our study was not powered to detect differences in outcomes between the two countries, and further studies are thus needed to explore the apparent differences in serological responses seen following antibiotic treatment.

A strength of our work is the use of baseline PCR to stratify participants. This method allows us to explore clinical and serological cure rates in both active yaws (in *T pallidum* ssp *pertenue* PCR-positive lesions) and presumed latent yaws (seropositive but PCR-negative for *T pallidum* ssp *pertenue*). PCR analyses allowed us to provide prospective data showing that both standard-dose and low-dose azithromycin were 100% effective for the clinical cure of skin ulcers caused by *H ducreyi*, which has emerged as a major cause of ulcerative lesions among children in tropical settings.^4,24,25^ The proportion of participants achieving clinical cure was also very high in lesions for which baseline PCR was negative for both *T pallidum* ssp *pertenue* and *H ducreyi*. This finding might reflect effective treatment of other ulcers caused by other azithromycin-susceptible pathogens, such as *Streptococcus* spp and *Staphylococcus aureus*, or healing of self-limiting skin lesions. Overall, the clinical effects of azithromycin seen in this study indicate that mass treatment with azithromycin is likely to provide additional benefit beyond its effect on yaws, which should be helpful for community acceptability. Our data also provide reassurance that the efficacy of low-dose azithromycin is not significantly different to standard-dose azithromycin for the serological cure of individuals with presumed latent yaws.^29^

A major limitation of this study is the smaller than anticipated proportion of individuals with active yaws who were enrolled in the study. The percentage of participants with *T pallidum* ssp *pertenue* PCR-positive lesions was slightly lower than that reported in some previous studies.^3,4^ Although this discrepancy might reflect a variation in the underlying causes of skin ulcer disease in the current study, it might also reflect issues with the ulcer swabbing technique applied to lesions.

Although a significant number of participants did not achieve the per-protocol definition of serological cure by 6 months, several factors related to interpretation of treponemal serology should be considered. First, amongst participants not achieving serological cure, only one patient had a four-fold increase in RPR titre, consistent with definitive serological failure, while three other patients had two-fold increases in RPR titre during follow-up. We cannot exclude the possibility that reinfection occurred during the period of follow-up, which might explain the rises in RPR titre in these patients. The absence of mutations conferring azithromycin resistance at baseline or emerging during follow-up in these patients is reassuring. All other participants not achieving the per-protocol definition of serological cure during the trial did achieve clinical cure but had either no change in titre or a two-fold RPR titre decrease from baseline to 6 months. It can take at least 12–24 months for the full amplitude of the RPR titre decrease to be expressed after successful treatment, particularly in latent yaws.^29,30^ Extending the follow-up period to 12 months or longer could have increased the proportion of patients achieving serological cure, particularly in participants with presumed latent disease. Second, it is possible that some individuals classified as having latent infection at baseline (positive serology with negative lesion PCR for *T pallidum* ssp *pertenue*) and who had no significant change in RPR titres at 6 months were actually serofast (ie, individuals with a persistent low titre of non-treponemal antibodies following successful treatment). Unfortunately, since there are no tests available that can distinguish serofast status^31^ from true latent infection, it is not possible to accurately determine the effect of this phenomenon on the proportion of our *T pallidum* ssp *pertenue* PCR-negative cases who achieved serological cure.

In this RCT, low-dose azithromycin did not meet the non-inferiority criterion for serological cure among participants with PCR-confirmed active yaws, although non-inferiority was shown within the study population overall. In both study groups, the clinical cure rate was close to 100% and only one patient had definitive serological failure following treatment. Although further data are needed to unequivocally establish the non-inferiority of low-dose azithromycin, the data in this study, together with existing observational data, suggest that 20 mg/kg of azithromycin is an effective treatment for yaws. In co-endemic countries planning mass drug administration for trachoma, the current results provide reassurance that this will also have a beneficial effect on the prevalence of yaws. When azithromycin is coadministered for trachoma, yaws, or both diseases, close monitoring of the effects and for the emergence of drug resistance in *T pallidum* ssp *pertenue* will be crucial.

## Supplementary Material

Appendix 1

Appendix 2

## Figures and Tables

**Table 1:** Participant characteristics at baseline

	Low-dose group (n=292)	Standard-dose group (n=291)
**Country**		
Ghana	201 (69%)	199 (68%)
Papua New Guinea	91 (31%)	92 (32%)
**Age (years)**		
Median (IQR)	10·0 (8·0–12·0)	10·0 (8·0–12·0)
**Sex**		
Male	204 (70%)	208 (71%)
Female	88 (30%)	83 (29%)
**Number of lesions**		
Unknown	0	2 (1%)
1	181 (62%)	180 (62%)
2	63 (22%)	63 (22%)
3	20 (7%)	20 (7%)
4	28 (10%)	26 (9%)
**Lesion type (of the largest lesion)**		
Unknown	1 (<1%)	1 (<1%)
Papillomas	51 (17%)	60 (21%)
Ulcer	240 (82%)	230 (79%)
**Lesion location (largest lesion)**		
Unknown	1 (<1%)	1 (<1%)
Face and neck	20 (7%)	15 (5%)
Trunk	1 (<1%)	2 (1%)
Back	4 (1%)	10 (3%)
Arm	27 (9%)	26 (9%)
Leg	239 (82%)	237 (81%)
**Serology at baseline**		
Unknown	7 (2%)	3 (1%)
RPR <1:16	129 (44%)	131 (45%)
RPR ≥1:16	156 (53%)	157 (54%)

Data are number of participants (%), unless otherwise indicated. RPR=rapid plasma reagin.

**Table 2:** Trial outcomes in participants with PCR-confirmed active yaws

	Low-dose group	Standard-dose group	Difference between groups, % (95% CI)*
**Overall**			
Clinical and serological cure at 6 months	61/76 (80·3%)	68/81 (84·0%)	3·7% (–8·4 to 15·7)
Clinical cure at 4 weeks	76/76 (100·0%)	81/81 (100·0%)	0% (–3·3 to 3·5)
Serological cure at 6 months	61/76 (80·3%)	68/81 (84·0%)	3·7% (–8·4 to 15·7)
**Ghana**			
Clinical and serological cure	46/60 (76·7%)	53/64 (82·8%)	6·1% (–8·1 to 20·2)
Clinical cure at 4 weeks	60/60 (100·0%)	64/64 (100·0%)	0% (–4·2 to 4·4)
Serological cure at 6 months	46/60 (76·7%)	53/64 (82·8%)	6·1% (–8·1 to 20·2)
**Papua New Guinea**			
Clinical and serological cure	15/16 (93·8%)	15/17 (88·2%)	–5·6% (–26·6 to 17·2)
Clinical cure at 4 weeks	16/16 (100·0%)	17/17 (100·0%)	0% (–14·3 to 14·9)
Serological cure at 6 months	15/16 (93·8%)	15/17 (88·2%)	–5·6% (–26·6 to 17·2)

Data are n/N (%), unless otherwise specified.

* Percentage in standard-dose group minus percentage in low-dose group.

**Table 3:** Trial outcomes in prespecified secondary study populations

	Low-dose group	Standard-dose group	Difference between groups, % (95% CI)*
**All yaws serology-positive participants†**			
Clinical and serological cure	134/200 (67%)	132/206 (64%)	−3% (−12·1 to 6·3)
Clinical cure at 4 weeks	200/200 (100%)	204/206 (99%)	−1% (−2·8 to 0·9)
Serological cure at 6 months	134/200 (67%)	133/206 (65%)	−2% (−11·6 to 6·8)
**Yaws serology-positive, *Treponema pallidum* ssp *pertenue* PCR-negative at baseline**			
Clinical and serological cure	73/124 (59%)	64/125 (51%)	−8% (−19·8 to 4·7)
Clinical cure at 4 weeks	124/124 (100%)	123/125 (98%)	−2% (−4·6 to 1·5)
Serological cure at 6 months	73/124 (59%)	65/125 (52%)	−7% (−19·0 to 5·5)
**Yaws serology-positive, *T pallidum* ssp *pertenue* PCR-negative, and *Haemophilus ducreyi* PCR-positive**			
Clinical cure at 4 weeks	51/51 (100%)	51/51 (100%)	0% (−5·2 to 5·2)
**Yaws serology-positive, *T pallidum* ssp *pertenue* PCR-negative, and *H ducreyi* PCR-negative**			
Clinical cure at 4 weeks	73/73 (100%)	72/74 (97%)	−3% (−7·7 to 2·5)

Data are n/N (%), unless otherwise specified.

* Percentage in standard-dose group minus percentage in low-dose group.

† All participants with positive serology for yaws (*T pallidum* particle agglutination and rapid plasma regain tests) regardless of the result of their baseline lesion PCR.

## Research in context

### Evidence before this study

We searched PubMed using the terms “yaws”, “treponema”, “pallidum pertenue”, and “azithromycin”, without date or language restrictions. Trials conducted in both Papua New Guinea and Ghana have shown that single-dose 30 mg/kg (maximum 2 g) azithromycin is an effective treatment for yaws. A lower dose of azithromycin (20 mg/kg, maximum 1 g) is recommended by WHO for treatment of trachoma. Observational studies in countries that are co-endemic for trachoma and yaws have shown that mass administration of azithromycin for trachoma reduces the prevalence of both active and latent yaws in the community, but no previous prospective controlled trial has evaluated the relative efficacy of low-dose versus standard-dose azithromycin for the treatment of yaws.

### Added value of this study

We investigated the relative efficacy of low-dose and standard-dose azithromycin in the treatment of yaws. By stratifying the trial population on the basis of PCR positivity for bacteria in samples extracted from the patients’ lesions, we provide data on clinical and serological outcomes in active and latent yaws. In participants with active yaws, clinical cure occurred in a similar proportion of participants following treatment with low-dose and standard-dose azithromycin. In participants with active yaws who received low-dose azithromycin, the proportion who achieved serological cure (and therefore the composite primary outcome) did not meet the non-inferiority margin. There was a lower than anticipated serological cure response at 6 months, which reduced the overall power of the study. In participants with presumed latent yaws, serological cure responses did not differ between the treatment groups. Our findings, considered alongside existing observational data, imply that standard-dose and low-dose azithromycin are likely to be equally effective for patients with yaws.

### Implications of all the available evidence

20 mg/kg azithromycin seems to be effective in treating yaws. Due to the smaller than anticipated primary study population of *Treponema pallidum* ssp *pertenue* PCR-positive participants and the lower than expected serological cure response in both groups, further data are needed before low-dose azithromycin can be unequivocally adopted as the standard of care.
