# Supplementary material for: Comparative efficacy of low-dose versus standard-dose azithromycin for patients with yaws: a randomised non-inferiority trial in Ghana and Papua New Guinea
Source: Lancet Glob Health. Author manuscript; Available in PMC 2021 Mar 9. (PMC7116878; doi:10.1016/S2214-109X(18)30023-8)
Supplement: Appendix 1 [file EMS117777-supplement-Appendix_1.pdf]

THE LANCET
Global Health

Supplementary appendix

This appendix formed part of the original submission and has been peer reviewed. We post it as supplied by the authors.

Supplement to: Marks M, Mitjà O, Bottomley C, et al. Comparative efficacy of low-dose versus standard-dose azithromycin for patients with yaws: a randomised non-inferiority trial in Ghana and Papua New Guinea. *Lancet Glob Health* 2018; published online Feb 15. http://dx.doi.org/10.1016/S2214-109X(18)30023-8.

Comparative efficacy of low-dose versus standard-dose azithromycin on patients with yaws: a multi-country randomized controlled trial

Supplementary appendix

Examples of results of point-of-care test used in the trial **2**

Baseline RPR by country **3**

Serological Outcomes by baseline RPR **4**

Full table of adverse events **5**

Independent photo review **6**

Comparison of 4 week and 6 month outcomes for skin lesions **7**

<u>Examples of results of point-of-care test used in the trial</u>

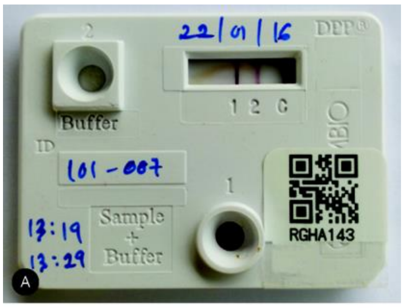

a) Dual positive point-of-care test

Both the treponemal (1) and non-treponemal (2) line are positive, consistent with current infection. This result was a criteria for study enrolment.

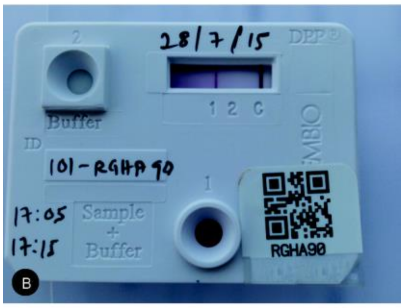

b) Treponemal line positive point-of-care-test

Only the treponemal line (1) of the test is positive. This is consistent with a previously treated treponemal infection.

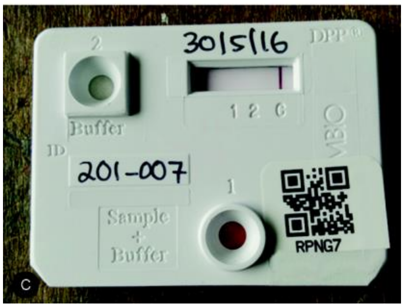

c) Negative point-of-care test

Both the treponemal (1) and the non-treponemal (2) lines are negative. This is consistent with no previous history of treponemal infection.

Baseline RPR by country

Quantitative RPR titre	Ghana n (%)	Papua New Guinea n (%)
0	41 (10.5%)	52 (28.6%)
1	16 (4.1%)	16 (8.8%)
2	17 (4.4%)	11 (6.0%)
4	25 (6.4%)	16 (8/8%)
8	38 (9.7%)	28 (15.4%)
16	54 (13.8%)	31 (17.0%)
32	104 (26.6%)	20 (11%)
64	75 (19.2%)	6 (3.3%)
128	21 (5.4%)	2 (1.1%)

Serological Outcomes by baseline RPR

	Baseline RPR	
**All Participants**	**<1:16**	**>=1:16**
Not Serologically Cured	65 (45.8%)	74 (28.0%)
Serological Cure	77 (54.2%)	190 (72.0%)
**20mg/kg Trial Arm**		
Not Serologically Cured	34 (48.6%)	32 (24.6%)
Serological Cure	36 (51.4%)	98 (75.4%)
**30mg/kg Trial Arm**		
Not Serologically Cured	31 (43.1%)	42 (31.3%)
Serological Cure	41 (56.9%)	92(68.7%)

Full table of adverse events

	Trial arm	
	20 mg/kg	30 mg/kg
None	277	279
Abdominal discomfort	6	2
Diarrhoea	2	1
Dizziness	4	2
Headache	0	2
Itching	2	0
Nausea	0	4
Rash	1	0
Stomach upset	0	1
**Total**	292	291

Independent photo review

Following the first meeting of the Data and Safety Monitoring Board, it was requested that an independent review of clinical lesion photos from the baseline and 4 week visits be performed on a subset of participants for verification of classification of clinical cure at 4 weeks.

A 10% selection of enrolled patients from Ghana was generated by simple random selection. Photos taken at baseline and 4-week follow-up were identified for these participants from the trial database

The independent grader (Dr Stephen Walker, Consultant Tropical Dermatologist) was provided with the photos and the clinical definitions used (healed / partially healed / unhealed) in the study. The grader was blinded to clinical details including the field assessment of clinical outcome. The grader assessed each photo using the clinical criteria outlined in the study protocol and this was used to define each individual as meeting/not-meeting the pre-defined primary outcome of clinical cure at 4 weeks (healed or partially healed lesion).

Forty patients were selected using simple random selection. Adequate photos were available at both baseline and 4 week follow-up for 36 patients (90%). The clinical outcome assigned by the clinical team and the independent grader are shown below.

**Assessment of degree of healing**	**Assessment of primary outcome of clinical cure at 4 weeks**	**n**	**%**
Complete agreement	Met primary outcome of clinical cure	29	72.50%
Independent review better than field team	Met primary outcome of clinical Cure	2	5%
	Did not meet primary outcome of clinical cure	0	0%
Independent review worse than field team	Met primary outcome of clinical cure	4	10%
	Did not meet primary outcome of clinical cure	1	2.5%
Ungradable	N/A	4	10%

The protocol defined that the 4-week clinical outcome of clinical cure was the same for 35/36 (97.2%) of patients with available photos and 35/40 (87.5%) of all patients in this random selection.

Comparison of 4 week and 6 month outcomes for skin lesions

	Month 6 outcome					
DPP positive and TP-PCR +VE at baseline	**20 mg**			**30 mg**		
**Week 4 outcome**	Completely healed	Not completely healed	Lost to follow-up	Completely healed	Not completely healed	Lost to follow-up
Partially healed	3	2	0	4	0	1
Completely healed	78	0	5	88	0	3
No improvement	0	0	0	0	0	1
Missing data at 4 weeks	1	0	1	3	0	1
All DPP-positive patients regardless of baseline PCR						
	Completely healed	Not completely healed	Lost to follow-up	Completely healed	Not completely healed	Lost to follow-up
Partially healed	18	3	3	17	0	3
Completely healed	234	3	16	234	4	17
No improvement	0	0	0	1	1	1
Missing data at 4 weeks	8	0	1	4	0	1
DPP positive and TP-PCR -VE at baseline						
	Completely healed	Not completely healed	Lost to follow-up	Completely healed	Not completely healed	Lost to follow-up
Partially healed	15	1	3	13	0	2
Completely healed	156	3	11	146	4	14
No improvement	0	0	0	1	1	0
Missing data at 4 weeks	7	0	0	1	0	0

DPP positive and TP-PCR -VE / HD PCR +VE						
	Completely healed	Not completely healed		Completely healed	Not completely healed	
Partially healed	9	1	1	4	0	1
Completely healed	59	2	3	58	1	11
No improvement	0	0	0	0	0	0
Missing data at 4 weeks	0	0	0	0	0	0
DPP positive and TP PCR -VE and HD PCR -VE						
	Completely healed	Not completely healed		Completely healed	Not completely healed	
Partially healed	6	0	2	9	0	1
Completely healed	97	1	8	88	3	3
No improvement	0	0	0	1	1	0
Missing data at 4 weeks	7	0	0	1	0	0
